# Radon Environmental Health Literacy among Health Council Participants in Northeast Tennessee

**DOI:** 10.13023/jah.0703.05

**Published:** 2025-09-01

**Authors:** Kawther Al Ksir, Stacy Stanifer, Hadii M. Mamudu, Megan Quinn, Deborah L. Slawson

**Affiliations:** East Tennessee State University; University of Kentucky; East Tennessee State University; East Tennessee State University; East Tennessee State University

**Keywords:** Appalachia, environmental health literacy, radon, risk perception

## Abstract

**Introduction:**

Radon, a radioactive gas, poses significant health risks, including lung cancer, and is prevalent in indoor environments. Understanding radon testing behavior and Environmental Health Literacy (EHL) is crucial for individuals to take appropriate preventive measures.

**Purpose:**

This study assessed radon-related EHL among Health Council participants in seven Northeast Tennessee (NETN) counties and examined associations between sociodemographic factors and EHL levels. Understanding radon knowledge and behaviors within this influential group is critical, as they often serve as trusted public health advocates.

**Methods:**

A cross-sectional survey was conducted from September – November 2023 among Health Council participants across seven counties in NETN, assessing sociodemographic characteristics and radon EHL. Descriptive statistics and linear regression analyses were employed to examine associations between sociodemographic variables and radon EHL scores.

**Results:**

A total of 131 Health Council members participated in the study. Of these total participants, the majority of participants were homeowners (60.3%). While 80.2% of participants had heard of radon, only 12% had tested their homes for it. TV commercials were the primary source of radon information. Younger age (p<0.001), renters (p<0.001), and those of Hispanic ethnicity (p=0.033) were associated with decreased EHL scores.

**Implications:**

Study’s findings reveal disparities in radon knowledge, testing behavior, and EHL among Health Council participants in NETN. Tailored risk communication strategies considering demographic factors are essential to bridge the radon EHL gap. Collaborative efforts between public health agencies, policymakers, and community members are crucial to enhancing radon knowledge and testing uptake.

## INTRODUCTION

Radon is a naturally occurring radioactive gas that forms from the breakdown of uranium in soil and rocks. It can accumulate in homes and buildings, especially in areas with poor ventilation or specific geological features.^1^ Exposure to radon is the second leading cause of lung cancer after tobacco smoke and the leading cause of lung cancer among never-smokers in the United States (U.S.).^1^ Each year, approximately 21,000 people in the U.S. die from radon-induced lung cancer –– including about 2,900 never-smokers.^1^ The risk of lung cancer from radon exposure is estimated to be 10 to 20 times greater for persons who smoke cigarettes than for those who have never smoked.^2^

A growing body of literature has examined the sociodemographic and geographic factors associated with radon testing behavior. These include age,^3^ income, educational attainment,^4^ smoking,^5^ homeownership,^6^,^7^ having children in the household,^8^ and ethnicity.^9^ Geographic location is particularly important, as individuals living in areas with known radon potential or identified radon “hotspots” may have greater awareness and are more likely to engage in testing and mitigation.^9^,^10^ A “radon hotspot” refers to an area where there is a high potential for elevated indoor radon levels.^1^ However, awareness and perceived risk alone do not always translate into protective behaviors.^11^ Studies show that individuals with higher radon knowledge and stronger perceptions of risk are more likely to test for radon and support mitigation efforts.^11^,^12^

In Tennessee (TN), and particularly in Northeast Tennessee (NETN), radon presents a significant public health concern due to the region’s geological composition, which includes uranium-rich formations such as limestone, shale, and phosphate.^13^ These geologic conditions, combined with variations in soil, building structures, and climate, create a landscape where radon exposure risk is uneven but potentially high across many communities.^14^,^15^ Climate and weather conditions significantly influence radon levels in buildings. Changes in temperature, atmospheric pressure, and soil moisture, particularly during colder months, can lead to higher radon concentrations indoors.^1^ The lack of a comprehensive, statewide radon policy further complicates mitigation efforts, leaving awareness and action largely dependent on individual knowledge and local engagement.^16^

Environmental Health Literacy (EHL) has recently emerged as a distinct subdiscipline that integrates core principles and methods from health literacy, risk communication, environmental health sciences, communication research, and safety culture.^17^–^20^ EHL is a key framework for understanding how individuals and communities interpret environmental health risks and take action to protect themselves. It encompasses the knowledge, skills, and competencies necessary to understand environmental health information and use it to make informed decisions.^21^ In the context of radon exposure, EHL is critical because it directly influences whether individuals recognize radon risk, seek testing, and pursue mitigation. Previous research has demonstrated the utility of EHL in shaping public responses to environmental hazards, particularly in underserved or rural communities where structural barriers to health are more pronounced.^22^–^24^

Within Appalachia, where socioeconomic disparities, lower health literacy, and environmental risk exposures intersect, EHL has been explored as a strategy to empower communities and bridge knowledge gaps.^25^ Studies have applied EHL frameworks to issues ranging from water contamination to air quality, highlighting the importance of culturally relevant, locally grounded approaches.^26^,^27^ However, few studies have specifically examined radon-related EHL in Appalachian populations, and even fewer have focused on community health leaders as key actors in disseminating environmental health information.^23^,^25^ This study builds on a limited but growing body of work using EHL as an outcome measure. We applied the Environmental Health Literacy Measure, a validated tool used in prior research to assess knowledge and confidence related to environmental risks,^28^ including studies on air and water quality in rural and community settings. By focusing on Health Council participants — individuals already engaged in public health initiatives — this study explores how EHL functions among a group uniquely positioned to influence radon awareness and action in their communities.

This cross-sectional study aimed to investigate associations between factors related to radon exposure and EHL among Health Council members in NETN. We hypothesized that Health Council participants with higher levels of education would demonstrate high levels of EHL given their health-related training and community involvement. This study also serves to fill a gap in the literature by exploring radon-related EHL within an Appalachian context and among Health Council Participants as local health influencers.

## METHODS

### Study Design

A cross-sectional study was conducted from September – November 2023 in NETN, including these seven counties: Carter, Greene, Hancock, Hawkins, Johnson, Unicoi, and Washington. A Health Council, known as a health coalition, is an organization that operates collaboratively.^29^ Their primary role is addressing public health concerns and advancing health and well-being. Comprising representatives from diverse community sectors, Health Councils play a pivotal role in promoting public health initiatives throughout TN.^29^ Health Council members were chosen for this study because they are trusted community stakeholders who play an active role in assessing local health needs, advising public health initiatives, and disseminating health information within their communities. Their health-related training and community leadership positions make them appropriate proxies for assessing environmental health literacy regarding radon, as they are often involved in educating others and shaping community health priorities. Residents of NETN who were 18 years or older, physically present in the U.S., and affiliated with one of the seven abovementioned NETN Health Councils were invited to participate in this study. A convenience sampling method was utilized for participant selection. A total of 131 participants completed the survey, which was administered via REDCap.^30^

### Recruitment

Participants were recruited through both in-person Health Council meetings and the NETN Health Councils’ regional listserv to ensure a well-rounded representation. The Principal Investigator (PI) attended council meetings held monthly in Carter County and bimonthly in Greene, Hancock, Hawkins, Johnson, Unicoi, and Washington Counties to introduce the study and invite participation. With permission from the TN Department of Health, the survey was also distributed through the region’s weekly email newsletter from September – November 2023. Participants accessed the REDCap^30^ survey via a QR code or online link using a computer, phone, or other device. As an incentive, respondents could enter a drawing to win one of 50 free radon test kits by providing their email at the end of the survey; email addresses were collected separately and used solely for the drawing.

### Measurement

Demographic characteristics were collected, including age, sex, race, income level, education level, smoking in the house, county of residence, having children living in that address, and homeownership status.

The EHL Measure was used to determine the NETN Health Council’s participants’ radon EHL. The EHL is a 14-item measure with scores ranging from 0–14, with higher scores reflecting greater EHL related to radon as an environmental health threat. The measure is based on the Finn and O’Fallon EHL framework (2017), which outlines five levels of knowledge, ranging from recognizing an environmental health threat to creating strategies to prevent or mitigate exposure.^31^ The EHL measure is divided into seven themes:

Recognize Radon Exposure as a Potential Health Hazard *(3 items; scores range from 0*–*3)*
Understand the Ways in which Radon Exposure Occurs *(1 item; scores range from 0*–*1)*
Apply Understanding to Inform Preventive Action *(4 items; scores range from 0*–*4)*
Analyze Data to Assess Personal Exposure Risk *(2 items; scores range from 0*–*2)*
Evaluate Radon Mitigation Options *(1 item; scores range from 0*–*1)* Create a Plan for Reducing Radon Exposure *(3 items; scores range from 0*– *3)*
Willingness to educate the community about radon *(1 item; scores range from 0*–*1)*.

The EHL measure started with the question: “Have you ever heard about radon?”. The EHL assessment currently addresses the initial four components within the EHL framework (Basic understanding, Intermediate comprehension, Advanced interpretation, and Strategic decision making). To encompass the fifth element, which pertains to “Active Engagement and Advocacy”, the following question adapted from Binder et al.^22^ was added: “To what extent are you willing to participate in informing your community about radon?” Participants can express their answers on a 5-point Likert scale featuring the following options: *(1) Not willing at all, (2) Somewhat not willing, (3) Undecided, (4) Somewhat willing, (5) Extremely willing*. A score of 1 point was given to those who answered “Somewhat willing” or “Extremely willing”. The final EHL score ranged from 0–15, with a higher score indicating a better radon EHL.

The question “Where have you heard about radon?” was used to assess information sources,^32^ and the question “How concerned are you about radon being in your home?” with the following response options to assess the level of concern about radon: *(1) Not concerned, (2) Somewhat concerned, (3) Concerned, (4) Very concerned*.^32^ Those who answered “No” to the question “Have you ever tested your home for radon?” were asked, “Why have you not tested your home for radon?” to explore underlying motivations and barriers that may have contributed to the decision of not conducting radon testing in their homes.^32^

The final questionnaire (See Appendix – A) was pilot tested with 30 individuals from East Tennessee State University and the Health Council coordinator. Responses from these individuals were not included in the final data analysis but helped determine the duration needed to take the survey (10 min) before administering it to the larger study population.

### Ethical Considerations

The study proposal was submitted for ethical approval from the Institutional Review Board of East Tennessee State University (IRB#: c0823.25e-ETSU). Participants were informed that their participation was voluntary, and their consent was obtained before data collection. Participants were assured that their responses would not affect their chances of winning a free radon test kit. When reporting the results, data were aggregated to prevent the identification of individual participants.

### Variables

The dependent variable in this analysis was the Environmental Health Literacy (EHL) score, treated as a continuous variable ranging from 0–15. Independent variables included age, educational level, income level, indication of smoking in the house, county of residence, homeownership status, and the presence of children in the household, totaling eight variables.^8^

All independent variables were considered potential covariates and confounders.

Age was controlled for because it has been shown to be related to knowledge, awareness, and behaviors related to radon exposure.^33^ Research has shown that older individuals are more likely to undergo radon testing compared to younger age groups,^28^ so the variable age in the regression model was treated as a dichotomous variable with individuals categorized as either being 35 years old or older, or younger than 35 years old. Educational level was accounted for to determine the independent effects of other variables beyond educational differences. Income level was considered as a potential confounding factor to examine whether observed associations are influenced by income disparities. The mention of smoking in the house was accounted for because of its confounding and interaction with radon exposure in increasing lung cancer risk. Residential location (county) was controlled for as radon levels can vary geographically and homeownership status was considered due to variations in radon concentrations among different housing structures.^10^ Finally, the presence of children in the household was examined to assess its impact on radon-related behaviors and concerns.

### Statistical Methods

#### Data analysis

SPSS software version 29 (IBM, Armonk, New York, U.S.) was used to analyze data. Descriptive statistics, such as frequencies and percentages, were used to summarize the categorical demographic variables such as sex, race, income level, level of education, smoking in the house, county, having children living at the address, and homeownership status. Mean and standard deviation were provided for age. Overall mean EHL scores and associated standard deviations were provided. EHL means by themes and by county and their corresponding standard deviations were reported. A one-way ANOVA was conducted to compare mean EHL scores across counties. To determine factors that predicted higher EHL scores, simple and multiple linear regression were used with EHL score as the outcome variable and the demographic variables mentioned previously as the predictor variables. The final multiple linear regression model controlled for any potential confounding. Unadjusted and adjusted beta values and corresponding p-values were reported for the simple and multiple linear regression analyses, respectively. The *p*-value significance threshold was set to 0.05.

## RESULTS

The average age of the 131 participants was 41.09 years (SD±13.8; Table 1). Participants identified as 17.6% female, while only 2.3% identified as male. Notably, a significant proportion, 80.2%, did not provide sex information. Participants were predominantly non-Hispanic (96.2%), white (91.6%). Geographically, participants were distributed across different counties, with Washington County representing the highest percentage at 58.8%. Homeownership status varied among participants, with the majority (60.3%) owning single-family homes. About 22.1% of participants reported having children in their households. Most participants had some post-secondary education, with 83.2% being college graduates. The majority (87.8%) reported a smoke-free home. Additionally, 54.9% of respondents reported annual household income levels of $75,000 or more.

Most of the study participants (80.2%) heard of radon, with TV commercials (25.2%) being the primary sources of information (Table 2). The largest percentage (47.3%) stated they were not concerned about radon being in their homes. Notably, 48.1% of participants cited a lack of knowledge on how to test as the primary reason for not testing their homes for radon. None of the respondents mentioned the presence of a radon mitigation system as a reason for not testing.

Overall, the mean EHL score was 4.92 (SD±3.5), suggesting a low average level of EHL (Table 3).

Table 4 presents the EHL score by county. A one-way ANOVA indicated that mean EHL scores differed significantly by county, F(6, 124) = 4.44, p < .001. The data highlights variations in radon awareness across different counties.

### Regression Results

In the regression analysis (Table 5), age was significantly associated with EHL scores, with younger individuals (<35 years old) exhibiting lower scores compared to their older counterparts (β_unadjusted = −2.32, p < 0.001; β_adjusted = −1.45, p = 0.045). Similarly, being Hispanic was associated with significantly lower EHL scores both before and after adjustment (β_unadjusted = −3.45, p = 0.033; β_adjusted = −6.036, p = 0.003). Moreover, renting a home was consistently linked with lower EHL scores (β_unadjusted = −2.45, p < 0.001; β_adjusted = −2.481, p = 0.001), highlighting the socioeconomic influence on EHL. Conversely, residing in Carter County was associated with significantly higher health literacy scores compared to Washington County (β_adjusted = 2.669, p = 0.032), suggesting regional disparities in EHL scores among Health Council participants.

The reported analysis demonstrates that the overall model is statistically significant (R^2^ = 0.320, F (19, 11) = 2.753, p < 0.001), indicating that the combination of the included variables (age, race, ethnicity, county, homeownership status, educational level, smoking in the home, level of income, children in the home) collectively explain approximately 32% of the variance in the EHL score. Essentially, this means that predictors collectively contribute to about one-third of the differences observed in EHL scores among Health Council participants.

## DISCUSSION

Understanding the level of EHL of Health Council participants in NETN is a critical first step to creating and implementing public health programs aimed at improving radon testing in the region. This study identified significant disparities in radon EHL among Health Council participants. Consistent with prior studies,^(34,45)^ our findings highlight the predominance of homeowners among participants. However, the notably higher proportion of missing sex information (80.2%) in our sample compared to previous research highlights the need for improved data collection practices, which may affect the interpretation of EHL results and limit the ability to explore sex-based differences in radon awareness and behaviors.^36^,^37^

Understanding radon EHL among Health Council participants in NETN is a critical first step to creating and implementing public health programs to improve radon testing in the region. This study identified significant differences in radon knowledge and testing among Health Council participants. Even though 80.2% have heard of radon, only 12% tested their home for it. Although the majority of participants had heard of radon, awareness alone may not translate into preventive action. Lack of knowledge on how to test one’s home for radon was reported as the primary reason for not testing (48.1%). This aligns with previous research highlighting the need for improved public education and awareness campaigns.^38^ Interestingly, none of the respondents mentioned the presence of a radon mitigation system as a reason for not testing, suggesting a potential gap in understanding the role of mitigation systems in reducing radon exposure.^23^ Additionally, participants who reported having a radon mitigation system in their homes may have previously conducted a radon test, as installation of such systems typically follows detection of elevated radon levels, or they could have bought a home with a mitigation system already installed.^38^

Despite radon being a leading cause of lung cancer, a significant proportion of participants (47.3%) were not concerned about its presence in their homes. This lack of concern may be attributed to insufficient awareness campaigns or ineffective dissemination of information regarding radon’s health risks.^38^

While Health Council participants are actively engaged in health-related activities, they reported TV commercials––contrasting with other studies where real estate or home inspectors were cited as the primary sources.^31^ This finding highlights the need for targeted and diversified communication strategies, such as education and outreach, to reach Health Council participants, as suggested in previous research.^33^

Analysis of EHL scores revealed variations across different sociodemographic factors. Younger individuals, Hispanics, and renters exhibited lower EHL scores, indicating disparities in EHL scores that contribute to radon knowledge and testing. These findings corroborate previous research emphasizing the influence of socioeconomic factors on radon awareness and mitigation behaviors.^39^ Moreover, regional disparities in EHL scores were observed, with residents in Carter County demonstrating significantly higher scores compared to Washington County, suggesting the need for targeted interventions tailored to specific geographical contexts.^39^

The study hypothesized that Health Council participants would exhibit high levels of EHL scores, given their presumed engagement in health-related activities and initiatives. Contrary to the hypothesis, the mean EHL score among Health Council participants was found to be relatively low (4.92, SD=3.5) out of a potential score of 15, indicating a limited understanding of radon-related health risks and mitigation strategies, which contradicts previous research reporting a mean of 8.9 using the same measure.^23^ This finding challenges the assumption that participants actively involved in health-related initiatives would possess higher levels of EHL.

Several factors may contribute to the observed discrepancy between expected and actual EHL scores among Health Council participants. While participants may be engaged in health-related activities, these activities may not specifically focus on radon awareness and mitigation. As a result, participants may lack comprehensive knowledge and understanding of radon-related health risks. Moreover, participants may face barriers to accessing radon-related education and resources despite their involvement in the Health Council. Limited availability of targeted educational materials, radon testing kits, and support services within the community could hinder participants’ ability to acquire relevant knowledge and skills. This finding suggests that involving communities in the testing process can lead to greater knowledge regarding radon exposure reduction.

### Strengths and Limitations

To our knowledge, this study is the first investigation into radon EHL within the state of TN. By focusing on Health Council participants in TN, the study fills a critical gap in existing research, shedding light on the levels of radon knowledge among residents of this region. The findings of this study have important implications for public health policy and practice. Targeted educational campaigns aimed at increasing radon awareness and promoting testing should consider the diverse needs and preferences of the population, leveraging various communication channels and community resources. Furthermore, efforts to address barriers to radon testing, such as cost and knowledge gaps, should be prioritized through policy initiatives and targeted interventions.

This study intentionally focused on participants in Health Councils in NETN, and this population may be different from the general population in the region. Although Health Council members are influential community stakeholders, the use of a convenience sample limits the generalizability of these findings to the broader county populations. Future studies should include larger and more representative samples to validate these results and assess environmental health literacy across diverse community members. Finally, the small absolute number of Hispanic participants limits the generalizability of findings related to this subgroup, and the small number of people who reported their sex limits conclusions related to sex differences. Addressing these limitations in future research—particularly the overrepresentation of middle-class participants, which may not fully reflect the socioeconomic diversity of Northeast Tennessee— can enhance the validity and reliability of findings and contribute to a more comprehensive understanding of radon-related knowledge and behaviors.

## CONCLUSION

Our findings indicate lower EHL scores than expected. Therefore, a continuous assessment of radon EHL and the development of targeted interventions are needed. The disparity between awareness and concern regarding radon exposure emphasizes the need for tailored risk communication strategies that consider demographic characteristics and socioeconomic factors. These results indicate the importance of clearly defining the role of Health Council participants in sharing information about environmental hazards with the communities they represent. Moving forward, collaborative efforts between public health agencies, policymakers, and community stakeholders are essential to enhance radon awareness and promote testing uptake. Establishing clear radon regulations in TN, such as mandatory radon testing and disclosure during real estate transactions and setting enforceable action levels for mitigation, could strengthen public health protection and support community-level radon reduction efforts.

**SUMMARY BOX**

**What is already known about this topic?**

Radon exposure is a leading cause of lung cancer and is prevalent in certain geographic regions, including Northeast Tennessee. Environmental Health Literacy (EHL) is critical for promoting awareness and preventive behaviors such as home testing. However, public knowledge and testing rates remain low, and disparities exist across demographic groups.

**What is added by this report?**

This study is the first to assess radon-related EHL among Health Council members in Northeast Tennessee—individuals who serve as community health advocates. Findings identify specific sociodemographic factors—such as younger age, renting status, and Hispanic ethnicity—associated with lower EHL, as well as the dominance of TV commercials as the primary information source.

**What are the implications for future research?**

Future studies should explore targeted, culturally tailored communication strategies to address identified knowledge gaps and behavioral barriers. Research is also needed to evaluate interventions that leverage Health Council members as trusted messengers to improve radon awareness, testing uptake, and ultimately reduce radon-related health disparities.

## Supplementary Information



## Figures and Tables

**Table 1:** Sociodemographic Results (N=131)

		N (%) or Mean (SD)
**Age**		
		41.09 (13.80)
**Sex**		
	Male	3 (2.3)
	Female	23 (17.6)
	Missing	105 (80.2)
**Race**		
	White	120 (91.6)
	Black	5 (3.8)
	Asian	1 (0.8)
	American Indian	2 (1.5)
	Native Hawaiian	1 (0.8)
	More than one race	2 (1.5)
**Ethnicity**		
	Hispanic or Latino/Latina	5 (3.8)
	Not Hispanic or Latino/Latina	126 (96.2)
**County**		
	Carter	9 (6.8)
	Greene	15 (11.5)
	Hancock	3 (2.3)
	Hawkins	15 (11.5)
	Johnson	8 (6.1)
	Unicoi	4 (3.1)
	Washington	77 (58.8)
**Homeownership Status**		
	Apartment/Rental	17 (13)
	House/Rental	15 (11.5)
	House/Owner	79 (60.3)
	Condo/Owner	1 (0.8)
	Condo/Rental	2 (1.5)
	Other	1 (0.8)
**Having Children in the House**		
	Yes	29 (22.1)
	No	102 (77.9)
**Education**		
	Grade 12 or GED (High school graduate)	5 (3.8)
	College 1 year to 3 years (Some college or technical school)	17 (13)
	College 4 years or more (College graduate)	109 (83.2)
**Smoking in the House**		
	No	115 (87.8)
	Yes	16 (12.2)
**Income**		
	< $10,000	5 (3.8)
	$10,001 – $20,000	2 (1.5)
	$20,001 – $25,000	2 (1.5)
	$25,001 – $35.000	8 (6.1)
	$35,001 – $50.000	17 (13)
	≥$75.000	72 (54.9)

Most of the study participants (80.2%) heard of radon, with TV commercials (25.2%) being the primary sources of information (Table 2). The largest percentage (47.3%) stated they were not concerned about radon being in their homes. Notably, 48.1% of participants cited a lack of knowledge on how to test as the primary reason for not testing their homes for radon. None of the respondents mentioned the presence of a radon mitigation system as a reason for not testing.

**Table 2:** Awareness and Perceptions of Radon

		N (%)
**Have you ever heard of radon?**		
	Yes	105 (80.2)
	No	26 (19.8)
**Where have you heard about radon?**		
	Other (School, work, general, uncertain)	38 (29.0)
	TV commercial	33 (25.2)
	Internet	30 (22.9)
	TV program/news	26 (19.8)
	Family/Friend	23 (17.6)
	A realtor	16 (12.2)
	Newspaper/Magazine	10 (7.6)
	Radio commercial	8 (6.1)
	Radio program/news	7 (5.3)
	My doctor	6 (4.6)
**Have you ever tested your home for radon?**		
	No	82 (61.7)
	Yes	16 (12)
**How concerned are you about the possibility of radon being in your home?**		
	Not concerned	62 (47.3)
	Somewhat concerned	50 (38.2)
	Concerned	15 (11.5)
	Very concerned	4 (3.1)
		
**Why have you not tested your home for radon?**		
	I do not know how to test for radon	63 (48.1)
	Testing is too expensive	13 (9.8)
	I do not believe radon is a health threat to me or my family	11 (8.3)
	I do not own my home	11 (8.3)
	There is already a radon mitigation system in my home	0 (0)

**Table 3:** Environmental Health Literacy (EHL) Theme Scores (N=105)

Theme	Mean (SD)
Recognize Radon Exposure as a Potential Health Hazard *(3 items; scores range from 0–3)*	1.84 (1.1)
Understand the Ways in which Radon Exposure Occurs *(1 item; scores range from 0–1)*	0.46 (0.5)
Apply Understanding to Inform Preventive Action *(4 items; scores range from 0–4)*	1.36 (1.1)
Analyze Data to Assess Personal Exposure Risk *(2 items; scores range from 0–2)*	0.18 (0.4)
Evaluate Radon Mitigation Options *(1 item; scores range from 0–1)*	0.32 (0.5)
Create a Plan for Reducing Radon Exposure *(3 items; scores range from 0–3)*	0.22 (0.5)
Willingness to educate the community about radon *(1 item; scores range from 0–1)*	0.53 (0.5)
EHL score *(15 items; scores range from 0–15)*	4.92 (3.5)

NOTES: SD= Standard Deviation

**Table 4:** Environmental Health Literacy (EHL) Scores by County

County	N	Mean (SD)
Carter	9	6.5 (3.6)
Greene	15	5.09 (2)
Hancock	3	7 (0)
Hawkins	15	6.17 (2.9)
Johnson	8	5.2 (3)
Unicoi	4	7 (1)
Washington	77	6.26 (2.4)

NOTES: SD= Standard Deviation

**Table 5:** Sociodemographic Predictors of Environmental Health Literacy (EHL) Scores: Simple and Multiple Regression Results

		Simple linear regression		Multiple linear regression	
		β value (95% CI)	P value	β value (95% CI)	P value
**Age**					
	=>35 years old	Ref.	-	-	-
	<35 yeas old	−2.32 (−3.54, −1.09)	<0.001	−1.45 (−2.88, −0.03)	0.05
**Race**					
	White	Ref.	-	-	-
	Black	1.34 (−1.87, 4.54)	0.411	2.39 (−0.72, 5.50)	0.13
	Asian	−4.95 (−11.97, 2.07)	0.165	−3.17 (−9.80, 3.46)	0.35
	American Indian	2.62 (−2.38, 7.62)	0.301	2.64 (−1.96, 7.24)	0.26
	Native Hawaiian	−4.95 (−11.97, 2.07)	0.165	−6.36 (−14.30, 1.57)	0.12
	More than one race	−0.93 (−5.95, 4.09)	0.714	2.13 (−3.97, 8.23)	0.49
**Ethnicity**					
	Not Hispanic	Ref.	-	-	-
	Hispanic	−3.45 (−6.60, −0.29)	0.033	−6.04 (−9.92, −2.16)	< .01
**County**					
	Washington	Ref.	-	-	-
	Carter	1.52 (−0.90,3.94)	0.216	2.67 (0.24, 5.10)	0.03
	Greene	−0.28 (−2.21, 1.65)	0.774	1.02 (−0.89, 2.94)	0.29
	Hancock	−3.33 (−7.40, 0.75)	0.109	−2.34 (−6.46, 1.78)	0.26
	Hawkins	0.47 (−1.46, 2.40)	0.630		
	Johnson	0.47 (−1.46, 2.40)	0.630	0.70 (−1.13, 2.53)	0.45
	Unicoi	−0.84 (−3.41, 1.72)	0.517	0.66 (−1.86, 3.18)	0.60
**Homeownership Status**					
	Renters	−2.45 (−3.67, − 1.22)	<0.001	−2.48 (−3.95, −1.01)	< .01
	Owners	Ref.	-	-	-
**Education**					
	High school graduate	−1.78 (−4.98, 1.41)	0.272	0.37 (−3.00, 3.73)	0.83
	Some college or technical school	0.37 (−1.46, 2.20)	0.692	−0.04 (−1.80, 1.72)	0.96
	College graduate	Ref.	-	-	-
**Smoking in the Home**					
	No	Ref.	-	-	-
	Yes	−0.55 (−2.42, 1.33)	0.567	−0.39 (−2.29, 1.52)	0.69
**Income**					
	<$35,000	−0.51 (−2.34, 1.32)		0.88 (−1.14, 2.90)	0.39
	$35,001- <$100,000	Ref.	-	-	-
	≥ $100,000	0.65 (−0.92, 2.21)	0.414	−0.25 (−1.80, 1.31)	0.76
**Having Children in the Home**					
	No	Ref.	-	-	-
	Yes	−1.44 (−2.90, 0.02)	0.053	−0.91 (−2.37, 0.56)	0.22

NOTES: B = regression coefficient
