# Supplementary material for: Radon Environmental Health Literacy among Health Council Participants in Northeast Tennessee
Source: J Appalach Health. 2025 Sep 1;7(3):54–76. doi: 10.13023/jah.0703.05 (PMC12440301; doi:10.13023/jah.0703.05)
Supplement: Supplementary file 1 [file 7.3.5_AdditionalFile.docx]

## Appendix A: Study Questionnaire

1. Have you ever heard of radon?

- Yes
- No (**Skip to #21**)

1. What is radon?

- A man-made gas
- A naturally occurring gas
- A poisonous chemical
- I don’t know

1. Where have you heard about radon? (**Choose ALL that apply**)

- TV commercial
- Radio commercial
- TV program/news
- Radio program/news
- Newspaper/Magazine
- Internet
- My doctor
- Family/Friend
- A realtor
- Other (please specify) ……….

1. What disease does radon cause?

- Diabetes
- Lung cancer
- Pancreatic cancer
- I don’t know

1. If someone who smokes is exposed to high levels of radon, that person’s risk of developing lung cancer is …………………………. **compared to a non-smoker:**

- Increased
- Decreased
- About the same
- I don’t know

1. How does radon enter buildings?

- Cracks in basements, foundations, and/or walls
- Ventilation system
- Wood-burning stoves or other fuel-emitting sources
- I don’t know

1. How can you know if your home has high radon?

- Ask your neighbors
- Strong odor in the home
- Test your home
- Windows have a film
- I don’t know

1. How concerned are you about the possibility of radon being in your home?

- Not concerned
- Somewhat concerned
- Concerned
- Very concerned

1. Where do you test your home for radon?

- Anywhere as long as it is cool
- Attic or highest floor where radon rises
- Lowest level where you spend at least 8 hours per week
- I don’t know

1. Have you ever tested your home for radon?

- Yes
- No

1. When did you last test your home for radon?

- 0-2 years ago
- 3-5 years ago
- >5 years ago

1. Why have you not tested your home for radon? **(Choose all that apply)**

- I do not know how to test for radon
- I do not believe radon is a health threat to me or my family
- I do not have time to test
- Testing is too expensive
- I do not own my home
- There is already a radon mitigation system in my home
- Other (please specify): _______________________

1. Do you intend to test your home for radon?

- Yes
- No
- I haven't decided

1. Do you currently know your home’s radon level?

- Yes  What is your home's radon level in picocuries per liter (pCi/L)? ………………..
- No

1. At or above what level does the U.S. Environmental Protection Agency advise a person to take corrective action to lower indoor radon exposure?

- At or above 1.0 pCi/L
- At or above 4.0 pCi/L
- At or above 8.0 pCi/L
- I don’t know

1. What is the best way to protect yourself from radon exposure?

- Add an attachment on your furnace
- Install a ventilation-based radon mitigation system
- Open doors and windows
- Seal foundation and other cracks
- I don’t know

1. If your home has high radon levels, have you taken any steps to protect people in your home from radon exposure?

- Yes
- No
- I have tested in the past two years, and my radon is < 4.0 pCi/L
- I do not know my home radon level

1. If so, what steps have you taken?

- Added an attachment to your furnace
- Installed a ventilation-based radon mitigation system
- Opened doors and windows
- Sealed foundation and other cracks
- Other (please specify):  ………………………
- My radon is < 4.0 pCi/L
- I do not know my home radon level

1. If your radon is high and you have yet to take protective steps, when do you plan to do so? (Please select ONE)

- Never
- In the next year
- In the next 6 months
- In the next 3 months
- My radon is < 4.0 pCi/L and I plan to test every two years
- My radon is < 4.0 pCi/L
- I do not know my home radon level

1. To what extent are you willing to participate in informing your community about radon?

- Not willing at all
- Somewhat not willing
- Undecided
- Somewhat willing
- Extremely willing.

1. How do you define your sex?

- Male
- Female
- Other (Please specify) …...........

1. What is your age?  ………
2. Which of the following best describes your race? (**Select all that apply**)

- White
- Black or African American
- Asian
- American Indian/Alaskan Native
- Native Hawaiian or Other Pacific Islander
- More than one race

1. Which of the following best describes your ethnicity?

- Hispanic of Latino/Latina
- Not Hispanic of Latino/Latina

1. What is your highest level of education?

- Never attended school or only attended kindergarten
- Grades 1 through 8 (Elementary)
- Grades 9 through 11 (Some high school)
- Grade 12 or GED (High school graduate)
- College 1 year to 3 years (Some college or technical school)
- College 4 years or more (College graduate)

1. Do you or anyone else living in your home smoke cigarettes, cigars, or pipes?

- Yes
- No

1. What type of housing do you currently live in?

- Apartment/Rental
- Apartment/Owner
- House/Rental
- House/Owner
- Condo/Owner
- Condo/Rental
- Other ……………………….

1. What ZIP code do you currently live in?

| - 37601 | - 37602 | - 37604 | - 37605 |
| --- | --- | --- | --- |
| - 37614 | - 37615 | - 37616 | - 37640 |
| - 37641 | - 37642 | - 37643 | - 37644 |
| - 37645 | - 37650 | - 37656 | - 37657 |
| - 37658 | - 37659 | - 37680 | - 37681 |
| - 37682 | - 37683 | - 37684 | - 37687 |
| - 37688 | - 37690 | - 37691 | - 37692 |
| - 37694 | - 37711 | - 37731 | - 37743 |
| - 37744 | - 37745 | - 37765 | - 37809 |
| - 37810 | - 37811 | - 37818 | - 37857 |
| - 37869 | - 37873 | - Other |  |

1. How many years have you lived at your current address?

- Less than 5
- 5-10
- 11-15
- 16-20
- More than 20

1. Do you have children under 18 years of age living in your home?

- Yes
- No

1. Which best describes the total combined annual household income?

- Less than $10,000
- Less than $15,000 ($10,000 to less than $15,000)
- Less than $20,000 ($15,000 to less than $20,000)
- Less than $25,000
- Less than $35,000 If ($25,000 to less than $35,000)
- Less than $50,000 If ($35,000 to less than $50,000)
- Less than $75,000 ($50,000 to less than $75,000)
- Less than $100,000 ($75,000 to less than $100,000)
- Less than $150,000 ($100,000 to less than $150,000)?
- Less than $200,000 ($150,000 to less than $200,000)
- $200,000 or more

Thank you for completing the survey. If you are interested in entering a drawing to win a free radon test kit. Please provide your email address [here.](https://etsuredcap.etsu.edu/surveys/?s=TFP34TWFNEDKWWK9)
